# Robot-assisted low anterior resection after aluminum potassium sulfate and tannic acid sclerosing therapy for internal hemorrhoids

**DOI:** 10.1186/s40792-019-0715-5

**Published:** 2019-10-29

**Authors:** Yoshiro Itatani, Tomoaki Okada, Kenji Kawada, Koya Hida, Nobu Oshima, Susumu Inamoto, Rei Mizuno, Yoshihisa Okuchi, Yoshiharu Sakai

**Affiliations:** Department of Surgery, Graduate School of Medicine, Kyoto University, 54 Shogoin-Kawaharacho, Sakyo-ku, Kyoto, 606-8507 Japan

**Keywords:** Robot-assisted surgery, Internal hemorrhoid, Rectal cancer, ALTA, Low anterior resection, Double-stapling technique

## Abstract

**Background:**

Internal hemorrhoids are the most common anal diseases. Aluminum potassium sulfate and tannic acid (ALTA) injection is a new sclerosing therapy for the treatment of internal hemorrhoids. Although ALTA injection has been widely used, there are no previous reports of rectal cancer patients who underwent robot-assisted low anterior resection (Rob-LAR) after ALTA injection to treat internal hemorrhoids.

**Case presentation:**

A 70-year-old man with rectal cancer was presented to our hospital. He had an ALTA injection 2 months before presentation at a clinic due to hematochezia with internal hemorrhoids. The rectal tumor was located 7 cm above the anal verge, and Rob-LAR with the da Vinci Xi system was performed. The patient had sclerosis on the stump of the anal side, which made it difficult to transect the rectum with linear staplers. This required multiple repeats of compression through the SmartClamp feedback. After anastomosis with the double-stapling technique, we constructed a diverting ileostomy.

**Conclusion:**

Although ALTA injection is a promising strategy for internal hemorrhoids, rectal cancer should be excluded before the sclerosing therapy.

## Background

Internal hemorrhoids are the most common anal diseases. Conservative treatment such as ointments or suppositories containing steroids can be used to remove acute inflammation. However, sometimes the condition cannot be improved even with the conservative therapies implemented with lifestyle improvement, such as avoiding hard stools and straining to defecate. In such cases, injection sclerotherapy is a good candidate for treatment.

Aluminum potassium sulfate and tannic acid (ALTA; Zione, Mitsubishi Tanabe Pharma, Osaka, Japan) is a new sclerosing agent for internal hemorrhoids. ALTA injection has been widely accepted as a treatment option in Japan and is reported to be as effective as surgical resection [1, 2]. ALTA injection induces inflammation, resulting in reduced blood flow inside the internal hemorrhoids and relapse of the hemorrhoid cavity through secondary fibrosis caused by the inflammation [3]. ALTA sclerosing therapy is performed via so-called “4-step injections.” This requires skilled technique by injecting the upper, deeper middle, shallow middle, and lower parts of the submucosal space of the internal hemorrhoids [4]. Although ALTA injection resolves both prolapse and hemorrhage caused by internal hemorrhoids, if the injection is misplaced, ALTA damages tissues and causes complications such as ulcers and stenosis of the rectum [5].

Here, we present a case of a rectal cancer patient who underwent robot-assisted low anterior resection (Rob-LAR) using the da Vinci Xi system (Intuitive Surgical, Sunnyvale, CA) after ALTA sclerosing therapy for internal hemorrhoids.

## Case presentation

**Fig. 1:**
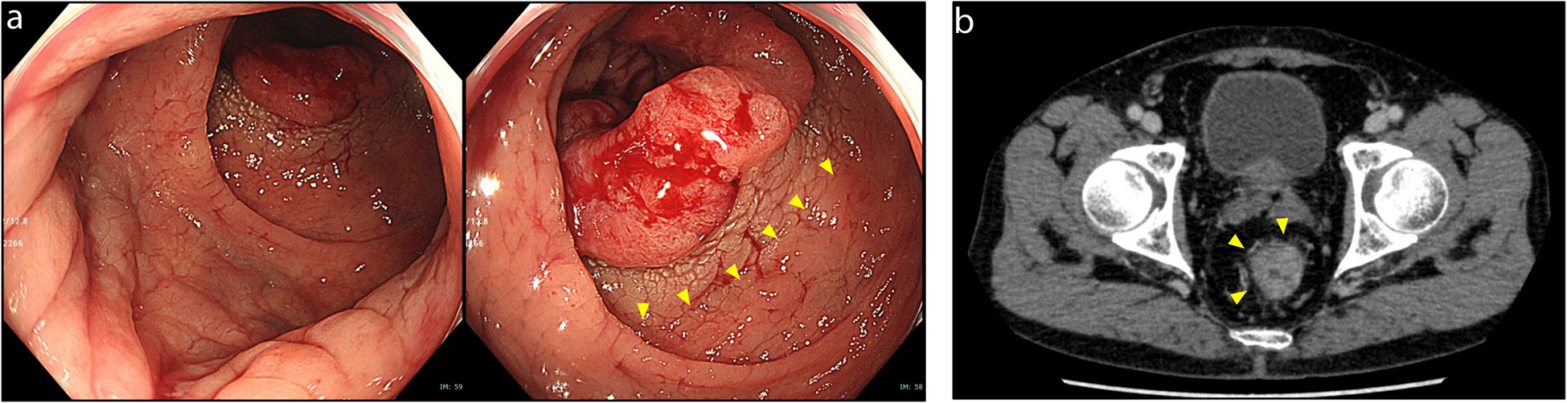
Preoperative evaluation of the patient. **a** Colonoscopy revealed a tumor 7 cm above the anal verge. Yellow arrowheads show edematous epithelium at the distal side of the tumor due to the ALTA sclerotherapy. **b** CECT showed thickening of the rectal wall in the lower rectum (yellow arrowheads)

A 70-year-old man was presented at our hospital with a rectal tumor. Two months earlier, he showed hematochezia and was diagnosed with internal hemorrhoids at a clinic. There he underwent sclerosing therapy with ALTA injection for his internal hemorrhoids. However, his hematochezia persisted after the ALTA sclerosing therapy, and his colonoscopy revealed a tumor in the lower rectum, 7 cm above the anal verge (Fig. 1a). Digital examination revealed the extension of the edematous rectum just below the tumor. Pathological examination of the rectal tumor indicated well-differentiated tubular adenocarcinoma. Contrast-enhanced computed tomography (CECT) identified thickening of the rectal wall in the lower rectum (Fig. 1b). Although there was no distant metastasis suspected, the most important problem in this case was the edematous sclerosis of the anus because of the ALTA therapy. This almost reached the distal side of the rectal tumor (Fig. 1c). Rob-LAR with diverting ileostomy was conducted to avoid severe complications due to the distal rectum sclerosis.

## Technique

**Fig. 2:**
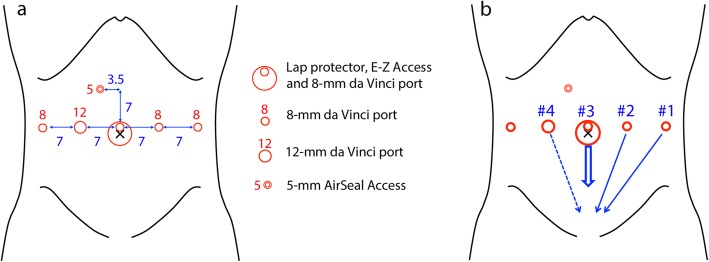
Schematic representation of the port placement. **a** A 3-cm longitudinal incision was placed on the umbilicus, and Lap Protector and E-Z access were inserted there. The first camera port (8-mm da Vinci port) was placed through E-Z access, and pneumoperitonium was started. Additional four da Vinci ports were placed symmetrically along the horizontal line of the umbilical incision with 7-cm intervals. A 5-mm AirSeal Access port was inserted in the right upper quadrant. **b** #1, fenestrated forceps; #2, fenestrated forceps with bipolar; #3, 0° camera; #4, monopolar curved scissors

**Fig. 3:**
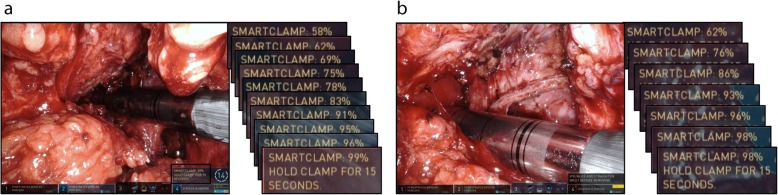
Division of the rectum with EndoWrist Staplers. **a**) The first staplers required 10 clamp-declamp cycles by the SmartClamp function. **b**) The second staplers required 7 clamp-declamp cycles by the SmartClamp function

Rob-LAR was performed using the da Vinci Xi system. The port placement is described in Fig. 2a and elsewhere [6]. The operator used two forceps connected to #1 and #2 arms with his left hand and one pair of monopolar curved scissors connected to #4 arm with his right hand (Fig. 2b). In doing so, even in the narrow pelvic space, the operator can widely expand the field with the #1 forceps and make an appropriate counter traction with the #2 forceps to dissect using the #4 monopolar scissors, without any interference between the arms. After reaching the pelvic floor, the distal side of the rectum was clamped with a Gutclamper (Kobe Biomedix, Kobe, Japan), and division of the rectum with an EndoWrist Stapler (30-mm green) was performed (Fig. 3). It required several attempts of clamp-declamp cycle by feedback from the SmartClamp function because of the thickening of the rectal wall caused by ALTA sclerotherapy. We used 3 linear cutting stapler cartridges, and a total of 17 clamp-declamp cycles were performed to achieve enough compression of the rectum for dissection. After removal of the specimen from the umbilical incision, end-to-end anastomosis with the double-stapling technique (DST, 25-mm EEA, Covidien, Dublin, Ireland) was performed. During DST anastomosis with the circular stapler, a total of 4 min waiting time (3 and 1 min before and after firing, respectively) was employed for secure stapling. To keep the anastomosis at rest and avoid severe complication related to the difficulty of rectal dissection, a diverting ileostomy was constructed.

## Result

**Fig. 4:**
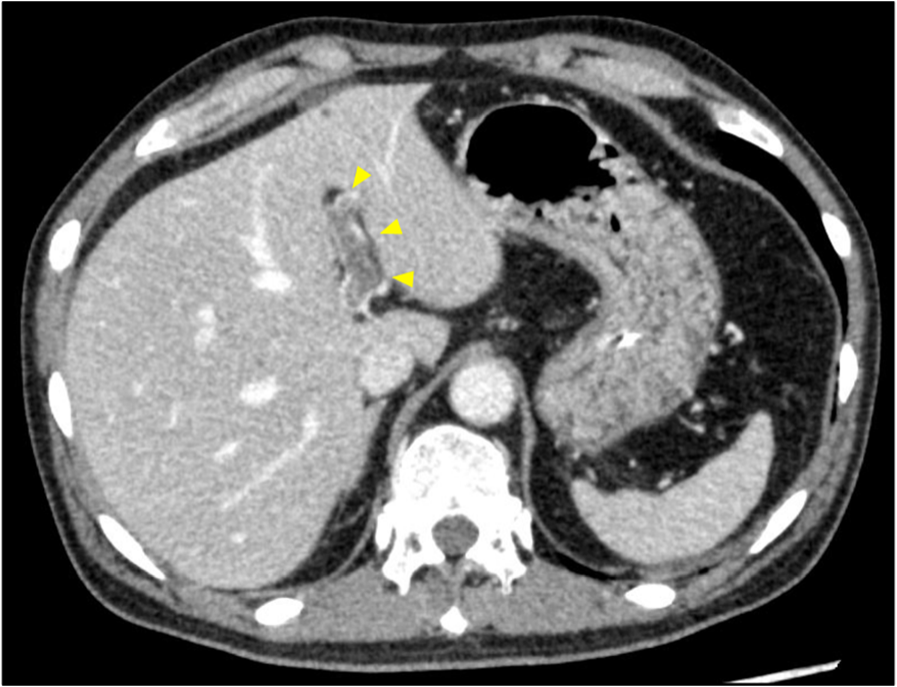
Postoperative CECT done at day 13 showed portal vein thrombosis (yellow arrowheads)

**Fig. 5.**
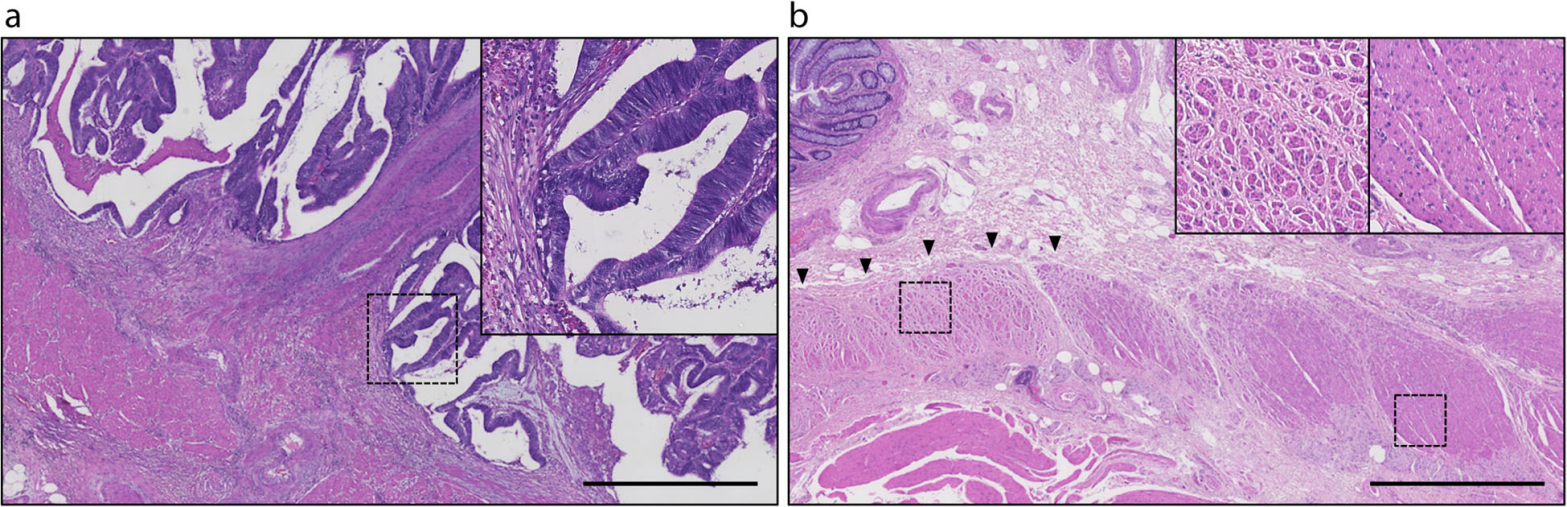
Hematoxylin and eosin staining of the specimen. Scale bar, 1 mm. **a**) Pathological examination of the tumor revealed well-differentiated tubular adenocarcinoma invading into muscularis propria layer. **b**) Histological examination of the distal stump showed significant fibrosis of the muscularis propria layer (black arrowheads and left small window) compared to the normal one (right small window)

The patients showed abdominal distension with elevated transaminases at day 13 postoperatively, and CECT revealed portal vein thrombosis (Fig. 4). It was unclear whether this complication was correlated with ALTA injection. Intravenous antithrombotic therapy with heparin was started, and the thrombosis disappeared after 7 days of antithrombotic therapy. Histological examination revealed well-differentiated tubular adenocarcinoma invading into the muscularis propria layer with no lymph node metastases (Fig. 5a). In addition, it also showed distinct fibrosis in the muscularis propria layer of the distal side of the tumor caused by ALTA injection (Fig. 5b). The diverting ileostomy was reversed 12 weeks after primary surgery.

## Discussion

Internal hemorrhoids are one of the most common diseases causing hematochezia. However, other severe diseases such as colorectal cancer and inflammatory bowel disease can also cause rectal bleeding. In most hemorrhoid cases, patients will not have rectal cancer. From the point of view of colorectal cancer diagnosis, comorbidity of hemorrhoids is the most common cause of missed opportunity for diagnosis of cancer [7]. Therefore the guidelines for the management of hemorrhoids published from the American Society of Colon and Rectal Surgeons recommend complete endoscopic evaluation of the colon in selected patients with symptomatic hemorrhoids and rectal bleeding, such as patients over 50 years old without complete examination within 10 years or those with positive fecal immunochemical testing [8, 9].

Therefore, it is extremely important to exclude the possibility that the patients with internal hemorrhoids also have a rectal tumor, both of which could be causes of hematochezia. However, even if patients do not have rectal tumors when treated with ALTA sclerotherapy, they could develop rectal tumors afterwards that require surgical treatment. Although little is known about the long-term pathological changes within the rectum after ALTA injection, the long-lasting success rate of ALTA sclerotherapy suggested that sclerosis after ALTA injection would be persistent [10]. In such cases, we have to pay close attention to dissect the sclerosed retcum. We routinely use Gutclamper before transection of the rectum for irrigation from anus to remove exfoliated tumor cells. In our case, the Gutclamper also helped to compress the distal side of the tumor, where ALTA injection caused sclerosis. The SmartClamp function of the da Vinci Xi system was very useful in achieving enough compression before dissection of the sclerosed rectum. It was reported that precompression or slow firing is important for secure stapling with a linear stapler [11, 12]. We applied this theory for DST anastomosis with a circular stapler, and a total of 4 min waiting time was employed to achieve sufficient compression with sclerosed rectum. Histological examination of the resected specimen revealed dramatic fibrosis in the muscularis propria layer at the stump even though ALTA was probably injected into the submucosal layer.

We previously reported 3 cases of laparoscopic rectal surgery for rectal cancer after ALTA therapy [13]. All of the 3 cases were planned to perform laparoscopic LAR with DST. However, only one case was successful with a diverting ileostomy. The other two cases were unsuccessful for DST anastomosis because of the difficulty to transect the sclerosed rectum with laparoscopic linear staplers. One of them had transanal hand-sewn anastomosis with a diverting ileostomy, and the other had Hartmann’s procedure. This case is the 4th case we experienced, and to our knowledge, it is the first reported case of Rob-LAR after ALTA sclerosing therapy. The patient in this case unfortunately suffered portal vein thrombosis after surgery, but in terms of anastomosis, the postoperative course was satisfactory and his diverting ileostomy was closed 12 weeks after primary surgery as scheduled without any complication.

## Conclusion

The presence of rectal cancer should be ruled out before injection sclerotherapy for internal hemorrhoids. Surgeons should be prepared to perform rectal cancer surgery in patients who have undergone sclerotherapy.

## Data Availability

Date sharing not applicable to this article as no datasets were generated or analyzed for this study.

## Abbreviations

ALTA: aluminum potassium sulfate and tannic acid
CECT: Contrast-enhanced computed tomography
DST: Double-stapling technique
Rob-LAR: Robot-assisted low anterior resection
